# Systematic evaluation of CrRNA design parameters for optimized Cas13d-mediated RNA targeting in chicken cells

**DOI:** 10.1007/s10142-025-01776-x

**Published:** 2025-11-26

**Authors:** Emily Hann, Debolina Majumdar, Daniel Layton, Mohamed Fareh, David M. Cahill, Mark Ziemann, Beata Ujvari, Karel A. Schat, Arjun Challagulla

**Affiliations:** 1 Australian Centre for Disease Preparedness, CSIRO Health and Biosecurity, Geelong, VIC Australia; 2 School of Life and Environmental Sciences, Deakin University, Geelong, VIC Australia; 3 Peter MacCallum Cancer Centre, Melbourne, VIC Australia; 4 Burnet Institute, Melbourne, VIC Australia; 5 Department of Microbiology and Immunology, College of Veterinary Medicine, Cornell University, Ithaca, NY USA

**Keywords:** CRISPR-Cas13, CrRNA design, RNA-targeting, Chicken, Mismatch tolerance

## Abstract

The CRISPR-Cas13 system has emerged as a powerful platform for programmable RNA targeting, offering efficient and sequence-specific silencing of coding and non-coding transcripts. The RNA-targeting capabilities of CRISPR-Cas13 have been harnessed to silence transcripts harbouring pathogenic mutations and combat infectious diseases. However, the molecular basis of on-target and collateral activity are not completely understood, limiting the utility of Cas13 systems. In this study, we delineate the principles for the development of effective crRNAs by targeting DsRed fluorescence reporter and synthetic influenza mRNA in chicken fibroblast DF1 cells. To systematically determine the optimal design for RfxCas13d crRNA, we investigated the minimum length of the crRNA, importance of protospacer flanking sequence, degree of mismatch tolerance, and off target effects. Our data reveal variable knockdown levels between crRNAs, in which several crRNAs achieved over 95% target knockdown. We show that crRNAs exhibit a high degree of tolerance to single-nucleotide mismatches, regardless of their position in the spacer sequence. However, 4-nt mismatches between the spacer and the target significantly reduces targeting efficacy, whereas eight nucleotide mismatches completely abolish the activity of RfxCas13d. Finally, we compared targeting efficiency and collateral activity of two widely used RfxCas13d and HfCas13d variants. Our data extend current understanding of Cas13d-mediated RNA targeting and offer a framework for rational crRNA design to enhance effectiveness in diverse applications, including antiviral strategies.

**Supplementary Information:**

The online version contains supplementary material available at 10.1007/s10142-025-01776-x.

## Introduction

The CRISPR-Cas system is an adaptive immune system of bacteria and archaea that has been harnessed for precise genome and transcriptome targeting applications (Andersson and Banfield 2008; Makarova et al. 2011; Jinek et al. 2012; Wang et al. 2020). CRISPR-Cas systems are broadly classified into two major classes, in which class 1 systems (types I, III, IV, and VII) encompass multi-subunit effectors, while class 2 systems (types II, V, and VI) encompass single multi-domain effectors (Shmakov et al. 2017; Wang et al. 2020). Given their simplicity and versatility, class 2 systems such as Cas9 (Type II) and Cas12 (Type V) have been harnessed for genome editing in a wide range of cell types and organisms. However, both Cas9 and Cas12, with an exemption of Cas12a2 (Dmytrenko et al. 2023), proteins are restricted to targeting DNA substrates exclusively. In contrast, the Cas13 proteins, which belong to the type VI of class 2 classification, are RNA-guided RNA-targeting proteins that mediate targeting of single-stranded RNA (ssRNA) in a sequence-specific manner (Abudayyeh et al. 2016, 2017). In recent years, several Cas13 subtypes have been identified, including Cas13a (Abudayyeh et al. 2017; Liu et al. 2017), Cas13b (Smargon et al. 2017), Cas13c (Huynh et al. 2020), Cas13d (Konermann et al. 2018), Cas13x and Cas13y (Xu et al. 2021), each of which exhibits distinct molecular mechanisms and targeting efficiency. Amongst the characterised proteins, the Cas13 protein derived from *Ruminococcus flavefaciens* (referred to as RfxCas13d) has emerged as an efficient and reliable RNA-targeting tool due to its compact size and high targeting efficiency (Konermann et al. 2018; H. Zhang et al. 2019a, b; reviewed in Yang and Patel 2024).

RfxCas13d functions as a two-component system, in which a 28 nt CRISPR RNA (crRNA) guides the Cas13d protein to its target site. The domain architecture of RfxCas13d comprises of a recognition lobe, which mediates crRNA binding, a nuclease lobe comprising of two HEPN domains, and a helical-2 domain. Upon target recognition, the crRNA-complexed Cas13d protein undergoes a spatial configuration that positions the HEPN domains in an outward-facing orientation, an arrangement that enables both target-specific (cis) cleavage and collateral (trans) cleavage of bystander non-target RNAs. These characteristic RNA targeting abilities of Cas13d effectors have been widely explored for the knockdown of both endogenous transcripts and exogenous RNAs, including RNA viruses. However, the rapid evolutionary dynamics of RNA viruses presents major obstacles for the design of crRNAs that retain targeting efficacy against mutations that emerge during viral evolution. For Cas13d-based antiviral strategies to be robust and broad-spectrum, a deeper understanding of the mechanistic parameters that govern Cas13d-mediated RNA knockdown, including the effects of crRNA length, protospacer flanking site (PFS) constraints, mismatch tolerance, and potential off-target cleavage is critical.

Although the collateral activity, a hallmark feature of various Cas13d proteins, has been harnessed for the development of diagnostic platforms (Gootenberg et al. 2017; Myhrvold et al. 2018), it remains a major barrier for intracellular RNA-knockdown and therapeutic applications, requiring high specificity. This is particularly a concern for in vivo applications, where pleiotropic RNA degradation induces unintended toxicity, developmental abnormalities, or potential embryonic lethality (Ai et al. 2022; Li et al. 2023; Shi et al. 2023). To address the collateral activity of RfxCas13d, Tong et al. (2023) developed an HfCas13d variant by site-directed mutagenesis of four amino acid substitutions (A134V, A140V, A141V, and A143V) within the HEPN-1 domain. Although HfCas13d protein has been shown to induce reduced collateral activity, growing evidence suggests that there is a trade-off between on-target activity and reduced collateral activity (Hart et al. 2025; Moreno-Sánchez et al. 2025). Moreover, it is critical to validate Cas13d effectors in species-specific cell lines, owing to the inherent differences in their transcriptomic profiles, RNA processing dynamics, and innate immune responses. These factors may also influence both the efficacy and safety of Cas13d-mediated RNA targeting.

Herein, we conducted a comprehensive molecular profiling to design effective RfxCas13d crRNAs by targeting the mRNA of DsRed, GFP, and synthetic influenza nucleoprotein (NP) in chicken cells. We systematically investigated key factors such as optimal crRNA length, PFS requirements, the influence of mismatches, mismatch tolerance, and collateral effects that may have a direct impact on knockdown efficiency. Our data indicate that RfxCas13d mediates potent on-target knockdown, in which variable knockdown levels were achieved based on the position at which spacers were designed to target and the type of modification introduced into the spacer sequence. Furthermore, we directly compared the extent of on-target and collateral activity of RfxCas13d and HFCas13d using a fluorescence knockdown assay and RNAseq analysis. The results obtained provide a roadmap for selection of effective Cas13d variants (i.e., Rfx and HF) and establish the design principles of effective spacers for RNA-targeting applications, including antiviral strategies.

## Materials and methods

### Cell culture

The chicken fibroblast DF1 cells (American Type Culture Collection number: CRL–12203) were maintained in Dulbecco’s Modified Eagle’s medium (DMEM), supplemented with 10% fetal bovine serum (FCS), 2mM L-glutamine, 10mM Hepes, 1.5% (w/v) sodium bicarbonate, 100 U/mL penicillin, and 100 µg/mL of streptomycin. Cells were incubated at 37 °C in 5% CO_2_.

### Plasmids

The RfxCas13d coding sequence was retrieved from Addgene Inc. (Plasmid No.: Plasmid #109049). A 3695 bp gene fragment containing Cas13d and Puromycin genes linked via an E2A linker was designed and chicken codon optimised using the IDT codon optimisation tool (Codon Optimization (idtdna.com). The restriction enzyme (RE) sites EcoRI site and XbaI were included at the 5’ end 3’ end, respectively, prior to ordering the vector from Integrated DNA Technologies (IDT, Singapore). The synthesised Cas13d-puro gene insert was cloned into pUC-Amp backbone by IDT. To generate the high fidelity RfxCas13d-Puromycin (referred to as HfCas13d) vector sequence, four A to V amino acid substitutions were made to the Cas13d sequence at sites defined by Tong et al. (2023) The resulting HfCas13d was fused to Puromycin (Cas13d-Puro), prior to ordering as synthesised transgene (IDT). The Tol2 transposon and Tol2 transposase (pTrans) vectors were a gift from Professor S. C. Ekker, Mayo Clinic Cancer Center, Rochester, MN, USA. To generate a Tol2 transposon vector carrying the Cas13d transgene, the synthesised RfxCas13d-Puro and HfCas13d were each cloned using the EcoRI and XbaI sites into the Tol2 transposon vector to generate Tol2-RfxCas13d and Tol2-HfCas13d, respectively (Fig. S1).

For expression of crRNAs, a crRNA expression vector was developed encoding a direct repeat, a human U6 promoter (hU6), and a BbsI RE site for crRNA cloning (Fig. S1). The DsRed expressing plasmid (pDsRed-Express, Catalogue. No. 632535) was purchased from Clonetech Laboratories (Mountain View, CA, USA). The eGFP expression plasmid was under the control of CAGGS promoter (referred to as pCAG-GFP). To generate the luciferase vectors encoding influenza nucleoprotein (NP) gene, a 200 bp sequence of the NP gene of A/turkey/Indiana/3703-003/2022 H5N1 strain was synthesised by IDT with XhoI and NotI RE sites at the 5’ and 3’ ends, respectively. The synthesised gene fragment was directionally cloned into the XhoI and NotI RE sites within siCheck-2 vector (CA#C8021).

### Design and cloning of CrRNAs into the expression vector

The crRNAs targeting the DsRed mRNA or GFP mRNA were manually designed to be 28 nucleotides in length, with either a C, T, U, or A nucleotide at the 3′ end as a PFS. Truncated crRNAs were designed by systematically reducing the length of nucleotides at the 3’ end to generate crRNAs of various lengths (i.e., 28 nt to 16 nt). To introduce mismatches into DsRed-specific crRNA, the crRNA sequence was mutated to contain either 1 nt, 2 nt, 3 nt, 4 nt, 8 nt, or 12 nt mismatches at either the 5’ end, the middle, or 3’ end. For targeting NP gene of H5N1, five crRNAs of 24 nt in length were designed against the positive sense of the A/turkey/Indiana/3703-003/2022 H5N1 NP gene. All crRNA sequences were oligo-synthesised with AAAC (top strand) or AAAA (bottom strand) and cloned into the BbsI RE site of the crRNA cloning vector and verified by Sanger sequencing (Micromon Genomics, Australia). All Cas13d crRNA sequences used in this study are provided in supplementary information (Supplementary Table 1).

### Generation of transgenic cell lines

To generate stable RfxCas13d and HfCas13d expressing cell lines, DF1 cells were seeded at a density of 1 × 10^6^ cells/well in a 6-well plate 24 h prior to transfection. Cells were co-transfected with 2 µg of appropriate Tol2-RfxCas13d or HfCas13d and 2 µg pTrans, complexed with 10 µL Lipofectamine 2000 (L2000) (Catalogue No. 11668019, Thermo Fisher Scientific, Waltham, MA, USA). To select for stable Cas13d expressing cells, transfected cells were maintained in media containing 1 µg/mL of puromycin (Catalogue No. A1113803, Thermo Fisher, Massachusetts, USA) for at least 14 days (Fig. S2).

### Fluorescence knockdown assays

For DsRed mRNA knockdown experiments, DF1-RfxCas13d or DF1-HfCas13d cells were seeded at a density of 2 × 10^5^ cells/well in a 24-well plate to produce a monolayer. The cells were transfected with 500 ng of non-targeting crRNA (crNT) or DsRed crRNA and 100 ng of DsRed expression plasmid complexed with 3 µL of L2000 reagent. For collateral activity assays, DF1-RfxCas13d or DF1-HfCas13d cells were seeded at a density of 2 × 10^5^ cells/well in a 24-well plate. Subsequently, 24 h post seeding, cells were transfected with 500 ng of appropriate crDsRed#2, 100 ng of pCMV-DsRed and 100 ng pCAG-GFP, and complexed with 3 µL of L2000 reagent. For GFP mRNA knockdown experiments, DF1-RfxCas13d or DF1-HfCas13d cells were seeded at a density of 2 × 10^5^ cells/well in a 24-well plate. The cells were transfected with 500 ng of crRNA (NT or GFP) and 100 ng of pCAG-GFP expression plasmid complexed with 3 µL of L2000 reagent. All knockdown experiments were performed at least twice independently, and the data presented represent one of the biological replicates.

Fluorescence knockdown was examined using fluorescence microscopy (Leica DMLB, Incucyte S3 Cell-analysis instrument) at 24 h post-transfection. To perform FACS analysis, cells were first harvested and resuspended in a FACS buffer (5% FCS in PBSA) before being analysed using a BD FACS LSRII (BD Biosciences, San Jose, CA, USA) for DsRed and GFP emission by utilising either the 488 nm and 513 nm lasers or 495 and 519 nm lasers, and appropriate detectors. To determine fluorescence knockdown, the proportion of DsRed or GFP positive cells and the DsRed or GFP mean fluorescence intensity (MFI), from three technical replicates were normalised to values for crNT. All graphs were generated using GraphPad Prism version 10.

### Luciferase knockdown assay

DF1-Cas13d cells were seeded at a density of 3.5 × 10^4^ cells/well in a 96-well plate to produce a monolayer. Cells were transfected with 90 ng of NP crRNA, or crNT, and 10ng of the NP-Luciferase plasmid complexed with 1 uL of L2000 reagent. At 24 h post-transfection, cells were prepared for luciferase reading using the Promega Dual-Luciferase^®^ Reporter Assay System (CA#E1910) as per manufacturer’s instructions. Luciferase activity levels were measured using the Biotek Synergy HT Microplate Reader. To quantify the firefly knockdown levels, firefly luciferase readouts for each NP crRNA, from five technical replicates, were normalised to values of crNT. All graphs were generated using GraphPad Prism version 10.

### RNA sequencing (RNAseq)

DF1-Cas13d or DF1-HfCas13d cells were seeded at a density of 2 × 10^5^ cells/well in a 24-well plate. Subsequently, 24 h post seeding, cells were transfected in triplicates with 500 ng of crDsRed#2, or crNT, 100 ng of pCMV-DsRed and 100 ng pCAG-GFP. Twenty-four hours post-transfection, RNA was extracted and purified from DF1 cells using RNeasy Plus Mini Kit (Qiagen) and the integrity of the RNA was measured using a 2100 Bioanalyser (Agilent). The RNA was sent to the Australian Genome Research Facility, Melbourne, Australia (AGRF) for RNA sequencing.

Skewer 0.2.2 (Jiang et al. 2014) was used to trim poor quality bases from the 3’ end of reads, with a phred threshold of 20. Kallisto v0.48.0 (Bray et al. 2016) was then used to map reads to the chicken transcriptome (Ensembl v113). Transcript read counts were then imported into R v4.4.2 and aggregated to gene level. Differential expression was conducted with DESeq2 v1.44.0 (Love et al. 2014) after removing genes with low counts (defined as < 10 counts per sample on average across all samples in the comparison).

Gene ontology terms were downloaded from Gene Ontology archive site (https://release.geneontology.org/2024-11-03/annotations/goa_chicken.gaf.gz) (The Gene Ontology Consortium et al. 2023) in GAF format and transformed to GMT format for pathway enrichment analysis. Enrichment analysis was conducted using the Mitch bioconductor package (Kaspi and Ziemann 2020). Genes and ontologies with false discovery rate corrected p-values (FDR) less than 0.05 were considered significant. RNA-seq data has been deposited to NCBI GEO and is available under accession number GSE305666 (https://www.ncbi.nlm.nih.gov/geo/query/acc.cgi?acc=GSE305666).

## Results

### Cas13d mediates high on-target knockdown in chicken cells

**Fig. 1:**
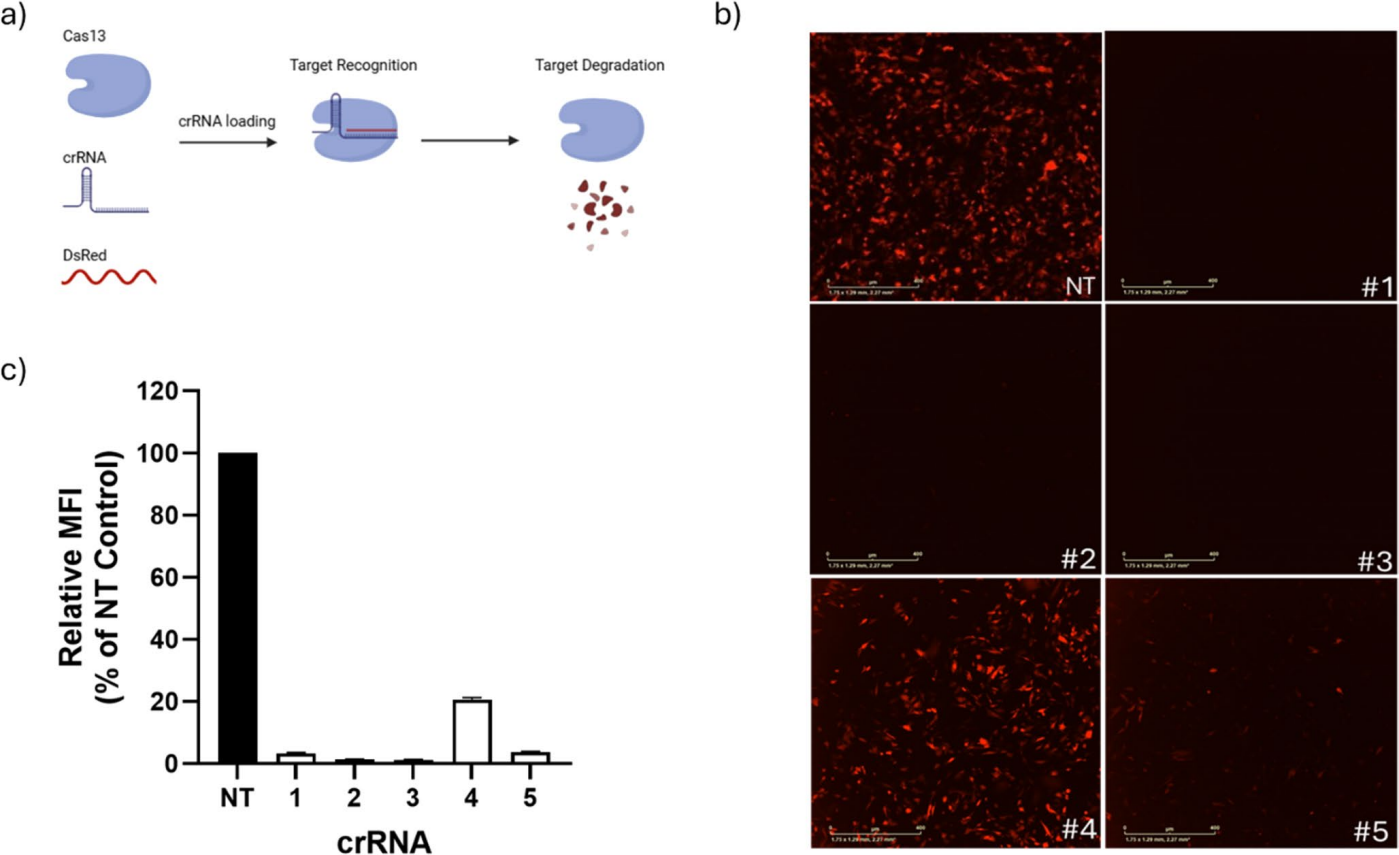
RfxCas13d-mediated knockdown of DsRed in chicken fibroblast DF1 cells. **a** Schematic of RfxCas13d-mediated targeted recognition and degradation of DsRed mRNA (**b**) Representative fluorescence microscopy images of RfxCas13d targeting of DsRed using five different crRNA in stable RfxCas13d-expressing DF1 cell line. **c** DsRed fluorescence knockdown determined by flow cytometry. Data points in the graph are averages of the normalised mean fluorescence intensity from technical triplicates. Error bars show SD

To establish RfxCas13d-mediated transcript knockdown (Fig. 1a), we first generated a chicken fibroblast DF1 cell line (DF1-RfxCas13d) by stably integrating the RfxCas13d-Puro transgene into the chicken genome using the Tol2 transposon system (Fig. S1 & S2). We selected the DsRed fluorescent reporter gene as our target and designed five different 28-spacer crRNAs (crDsRed#1–crDsRed#5) of 28-nt in length, each fully basepairing with unique regions of DsRed mRNA. The designed crRNAs sequences were then each cloned into a crRNA expression vector and expressed under the control of a U6 promoter (Fig. S1). To examine RfxCas13d-mediated DsRed knockdown, DF1-RfxCas13d cells were co-transfected with pCMV-DsRed vector and DsRed-crRNA vectors. Cells transfected with a non-targeting (NT) crRNA sequence (crNT) were used as a negative control. Following co-transfection, the knockdown of DsRed expression was examined by fluorescence microscopy and flow cytometry analysis 24 h post-transfection. Fluorescence microscopy analysis revealed that four out of the five DsRed-targeting crRNAs induced potent knockdown, in which DsRed fluorescence was almost undetectable. In contrast, cells transfected with the NT-crRNA vector did not show any DsRed fluorescence knockdown, confirming that RfxCas13d mediates DsRed knockdown in a spacer-dependent manner (Fig. 1b). Flow cytometry analysis revealed that DF1-RfxCas13d cells transfected with crDsRed#1, crDsRed#2, crDsRed#3, and crDsRed#5 exhibited strong and specific transcript suppression, in which DsRed knockdown by these four crRNAs reached 97% − 99% (Fig. 1c). However, crDsRed#4 did not show strong DsRed knockdown, suggesting that not all designed crRNAs are capable of inducing mRNA knockdown and that RNA secondary structures, the spatial accessibility of specific mRNA regions, or nucleotide composition of the spacer sequence may influence the knockdown efficiency.

### RfxCas13d-mediated knockdown is independent of PFS context

**Fig. 2:**
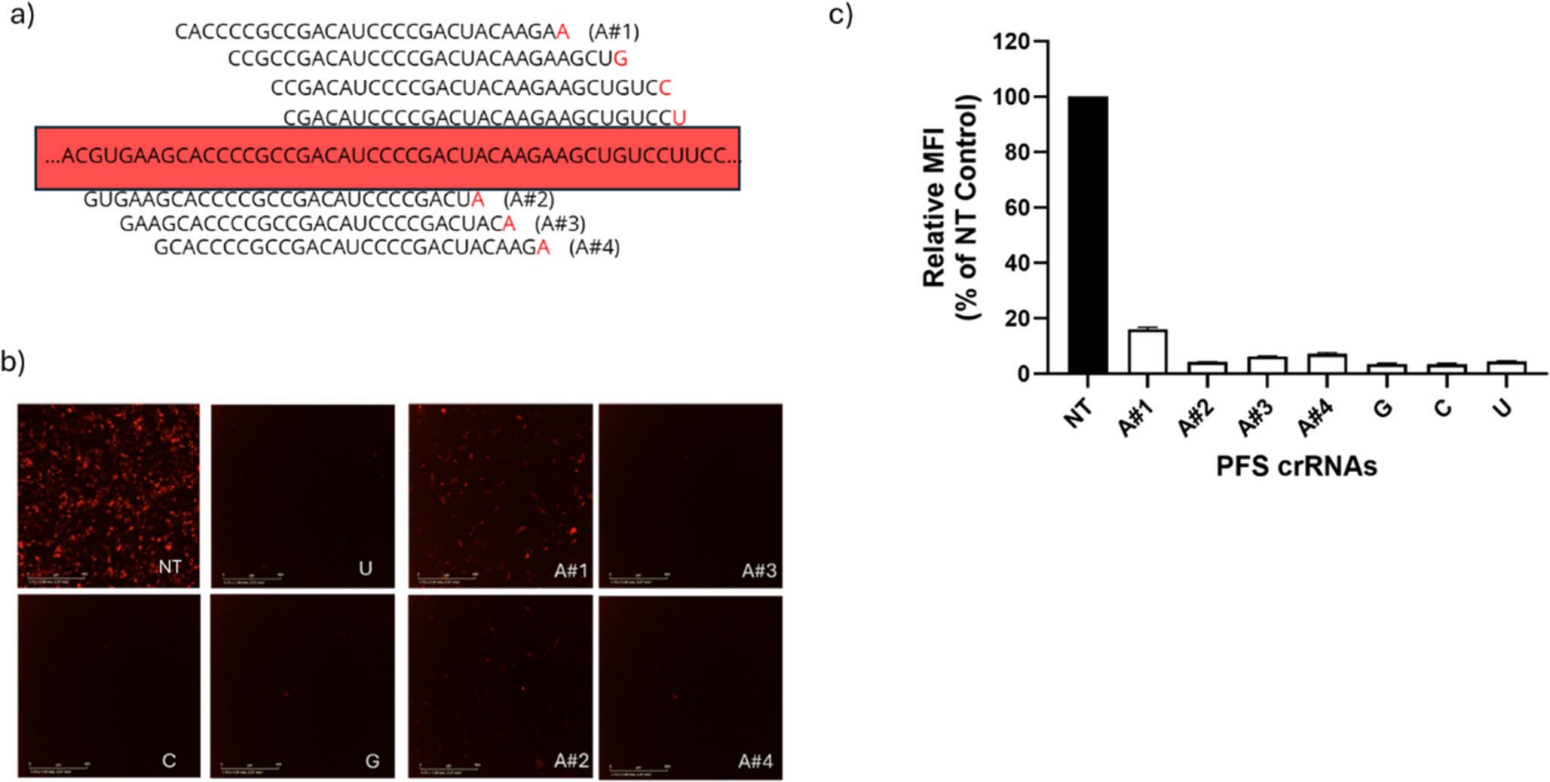
RfxCas13d mediates mRNA targeting in a PFS-independent manner. **a** Schematic of the DsRed mRNA and crRNAs with A, G, U and C PFS that were designed in proximity. **b** Representative fluorescence microscopy images of four A PFS and C, U and G nucleic acid crRNAs determined by FACS flow cytometry. **c** DsRed fluorescence knockdown by crRNAs with different PFS determined by flow cytometry. Data points in the graph are averages of the normalised mean fluorescence intensity from technical triplicates

To test whether RfxCas13d-mediated mRNA knockdown is dependent on PFS, we compared the knockdown efficiency of a previously validated crRNA (crDsRed#2), which contains a U PFS, with three newly designed crRNAs targeting adjacent regions of the DsRed transcript. These newly designed crRNAs possess A, C, and G PFS. respectively and are in spatial proximity to the crDsRed#2 target site (Fig. 2a, top panel). Fluorescence microscopy analysis revealed that crRNAs with C, G, or U PFSs exhibited a clear reduction in DsRed fluorescence. However, crRNAs possessing an A PFS did not result in comparable DsRed fluorescence knockdown (Fig. 2b).

To determine whether the failure to induce a robust DsRed knockdown by crRNAs with A PFS was due to PFS dependency or a positional effect, we performed a follow-up study by designing three additional crRNAs with an A PFS (Fig. 2a, bottom panel). The three new crRNAs with A PFSs were designed to bind spatially close to the previously validated target site (crDsRed#2). To examine the knockdown efficiency achieved by crRNAs with different PFS variants, we performed a comparative transfection experiment using all seven crRNAs: four with A PFS and three previously validated crRNAs with C, G, and U PFS, respectively. Flow cytometry on transfected cells revealed that three newly designed A PFS crRNAs (A#2, A#3, and A#4) exhibited robust knockdown of DsRed fluorescence, in which targeting efficiencies were ranged between ~ 92–95%. The knockdown levels achieved by these newly designed A PFS crRNAs are comparable to those observed with C (~ 96%), G (~ 96%), and U (~ 95%) PFS crRNAs, indicating that Cas13d-mediated knockdown does not rely on a strict PFS requirement for crRNAs (Fig. 2c).

### Cas13d mediates knockdown in a CrRNA length-dependent manner

**Fig. 3:**
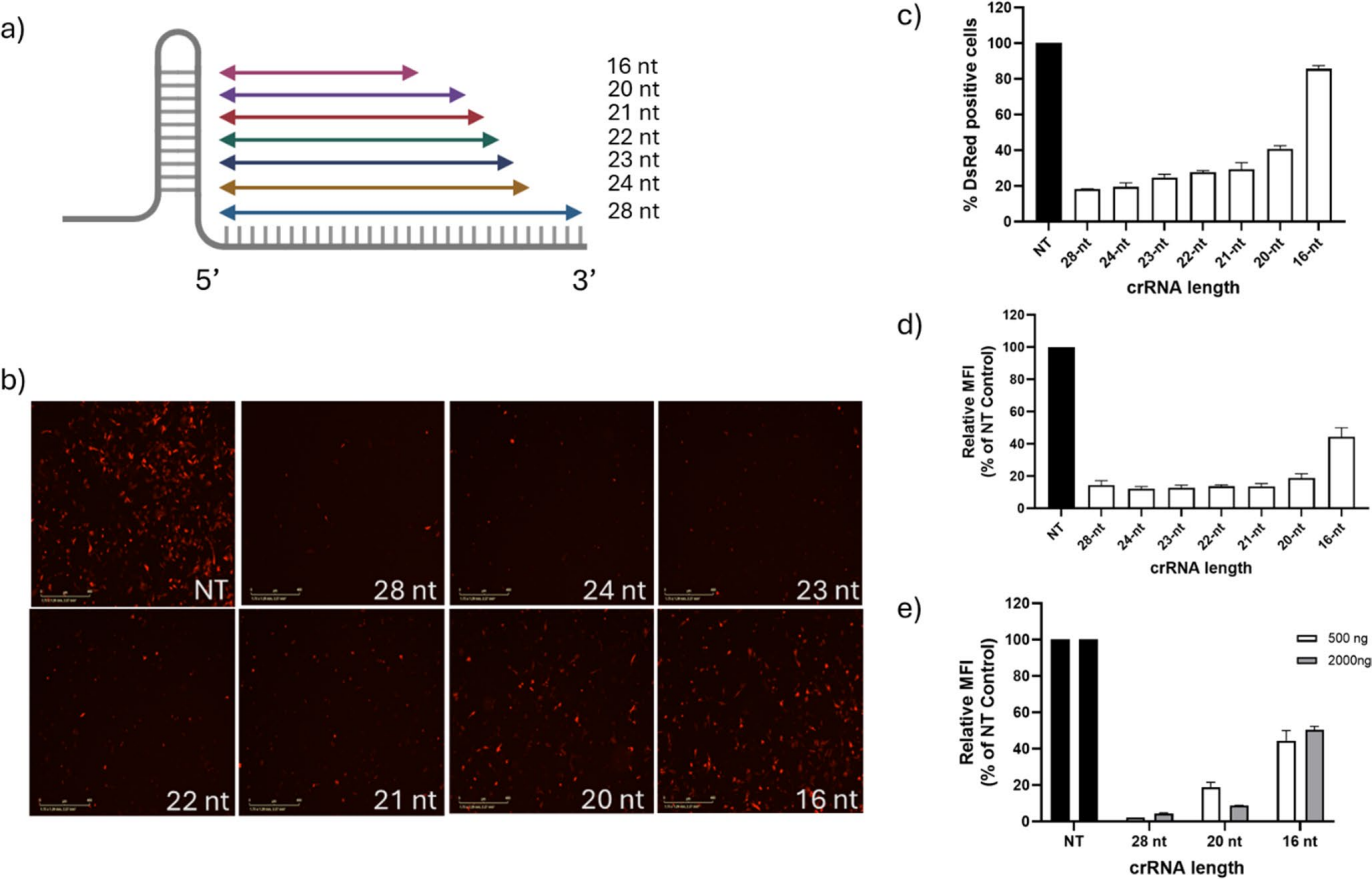
RfxCas13d-mediated mRNA targeting is crRNA length dependent. **a** Schematic of crRNA truncations performed for investigating minimal length required for Cas13d mediated mRNA cleavage. **b** Representative fluorescence microscopy images show the knockdown DsRed by different truncated spacers. **c** and (**d**) DsRed fluorescence knockdown by different spacers was determined by flow cytometry. (**e**) DsRed fluorescence knockdown of ineffective crRNAs (i.e., 20-nt and 16-nt) by increasing the plasmid concentration. Values shown as the mean ± Sd. Data points in the graph are averages of the normalised values from technical triplicates

RfxCas13d-mediated mRNA targeting typically uses crRNAs with a canonical spacer length of 28-nt. This spacer length has been shown to induce high targeting efficiency in various intracellular and biochemical knockdown studies. However, designing a 28-nt crRNA that enables coverage of multiple subtypes of rapidly evolving RNA viruses can be often challenging. One approach to circumvent these issues is by designing shorter spacers that can mediate similar levels of knockdown as the canonical spacer length of 28 nt. To test this, we systematically shortened the spacer region of the validated 28-nt crRNA (DsRed-crRNA#2) to generate a set of six truncated crRNAs (24–16 nt) (Fig. 3a). This rational spacer truncation was carried out at the 3’ end of the crRNA rather than at the 5’ end (where the PFS is present) to avoid affecting the Cas13d knockdown efficiency by the PFS. Following the co-transfection of chicken DF1-Cas13d cells with individual truncated crRNAs and the DsRed expression plasmid, the knockdown efficiency was evaluated by fluorescence microscopy and flow cytometry at 24 h post-transfection. Fluorescence microscopy examination of transfected cells indicated that truncated crRNAs of 24-nt, 23-nt, 22-nt, and 21-nt exhibited pronounced knockdown, in which the DsRed fluorescence knockdown was comparable to those observed with the full-length 28-nt crRNA (positive control). In contrast, cells transfected with the shorter 20-nt or 16-nt crRNAs displayed only modest reductions in DsRed signal relative to NT control cells (Fig. 3b).

For accurate quantification of knockdown efficiency, transfected cells were analysed by flow cytometry to determine: (i) the percentage of DsRed knockdown cells (Fig. 3c), and (ii) the DsRed fluorescence based on mean fluorescence intensity (MFI) (Fig. 3d). Flow cytometry analysis revealed that cells transfected with 28-nt and 24-nt crRNAs exhibited 80% knockdown of DsRed-expressing cells compared to NT transfected. The truncated 24-nt to 21-nt crRNAs showed a trend of decreasing targeting efficiency, with a 2–3% loss observed for every nucleotide truncation. However, DsRed knockdown using a 20-nt crRNA was greatly affected, with only ~ 60% reduction achieved. DsRed targeting was greatly diminished using 16-nt crRNA, suggesting a strong link between the knockdown efficiency and crRNA length (Fig. 3c). Similarly, quantitative analysis of DsRed MFI revealed that cells transfected with different lengths of DsRed crRNAs (28-nt to 21-nt) displayed up to ~ 90% DsRed knockdown, while cells transfected with 20-nt crRNAs exhibited ~ 85% knockdown efficiency (Fig. 3d). In contrast, DsRed knockdown efficiency was substantially affected in cells transfected with the 16-nt crRNA, with fluorescence levels reduced by only ~ 50%, indicating a profound loss of Cas13d targeting activity when using shorter crRNAs.

To assess whether increasing the plasmid concentration enhances the knockdown efficiency of 20-nt and 16-nt crRNAs, we performed additional transfection by increasing crRNA vector concentration by four-fold (2000 ng) relative to the standard (500 ng) during transfection. DF1-RfxCas13d cells transfected with 2000 ng of the 20-nt crRNA showed markedly improved knockdown efficiency by achieving up to 92% reduction in DsRed fluorescence. In contrast, even a four-fold increase of plasmid concentration did not yield any noticeable increase of knockdown efficiency using the 16-nt crRNA, suggesting that RfxCas13d activity is spacer length-dependent (Fig. 3e).

### Mismatches in the CrRNA spacer influence Cas13d mediated knockdown

Effective targeting of Cas13d requires the use of a crRNA that maintains near-perfect complementarity at the spacer-target interface. This requirement poses challenges when designing spacers against rapidly mutating RNA viruses, in which emerging mutations may reduce the binding affinity of crRNAs and subsequent cleavage activity. Therefore, a detailed understanding of Cas13d’s sensitivity to mismatches is essential for the development of robust and broadly effective RNA-targeting strategies.

**Fig. 4:**
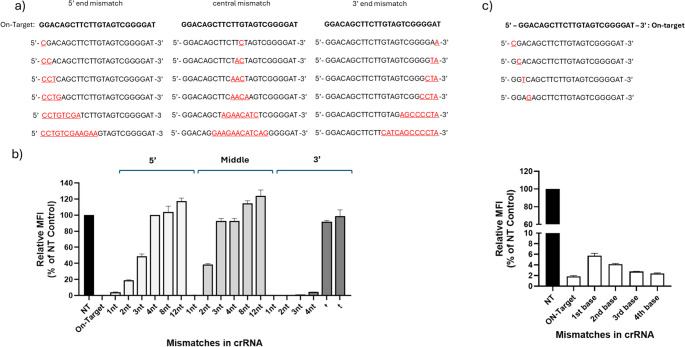
RfxCas13d-mediated mRNA targeting is sensitive to mismatches in the crRNA (**a**) List of designed spacers with introduced mismatches (1-nt, 2-nt, 3-nt, 4-nt, 8-nt or 12-nt) at 5’ end, middle and 3’end into the 24-nt function DsRed targeting spacer. The nucleotides highlighted in red are introduced mismatches The DsRed knockdown efficiency was compared between the various crDsRed with mismatches, on-target spacer (positive control) and crNT (non-targeting, negative control) (**b**) The percentage DsRed fluorescence knockdown by crRNAs with various introduced mismatches at 5’-end, central and 3’-end using flow cytometry. **c** Single-nt mismatches introduced at the 1 st, 2nd, 3rd and 4th bases from the 5’ end (near the PFS) of the spacer. The percentage DsRed fluorescence knockdown by flow cytometry. Values shown as the mean ± Sd. Data points in the graph are averages of the normalised mean fluorescence intensity from technical triplicates

To systematically evaluate the effect of mismatches on knockdown activity, we generated a panel of 18 crRNAs with unique mismatches into the spacer of the previous 24-nt DsRed-targeting crRNA. Specifically, 1-nt, 2-nt, 3-nt, 4-nt, 8-nt, and 12-nt mismatches were introduced into the 5′ end, central region, or 3′ end of the spacer (Fig. 4a). To test the knockdown efficiency, DF1-Cas13d cells were co-transfected with DsRed and crRNAs with different mismatches. The knockdown efficiency of mismatched spacers was compared to the 24-nt crRNA (positive control). Fluorescence microscopy images revealed that cells transfected with crRNAs with 1-nt mismatch were tolerated, but not with crRNAs containing four or more mismatches (Fig. S3 - S5). For accurate quantification of knockdown efficiency, transfected cells were analysed by flow cytometry to assess how different types of mismatches affected Cas13d knockdown activity. It was found that crRNAs with 1-nt mismatches at the 3’-end, middle and 5’-end were well tolerated with knockdown efficiency achieving ~ 96–99% knockdown, which was comparable to that of crRNA 24-nt (on-target) (Fig. 4b). However, the knockdown efficiencies varied for mismatches that are 2-nt or more, depending on the position at which they were introduced. For mismatches introduced at the 5′ end of crRNAs, a 2-nt mismatch resulted in approximately ~ 80% knockdown, while a 3-nt mismatch reduced knockdown efficiency to ~ 50%. Mismatches of 4-nt, 8-nt, or 12-nt completely abolished knockdown activity, indicating a tolerance threshold in this region. For the central region of crRNAs, mismatches of 2-nt significantly impaired knockdown with only ~ 60% knockdown activity. Introduction of 3-nt or more mismatches at this position completely abolished knockdown activity, suggesting the central region is highly sensitive to mismatches. In contrast, 3′ end mismatches were better tolerated in which knockdown efficiency remained largely unaffected up to 4-nt mismatches, indicating relative flexibility in this region. However, 8-nt and 12-nt mismatches at the 3′ end resulted in a complete loss of activity, reflecting an upper limit of toleration is maximum 4-nt at this position (Fig. 4b). Given that the 5’-end of the crRNAs is highly sensitive to mismatches, we conducted an additional study by designing four additional crRNAs with a single-nucleotide mismatch at the 1 st, 2nd, 3rd and 4th position (Fig. 4c). Flow cytometry analysis revealed a gradual recovery of knockdown activity similar to that of on-target as the mismatch is positioned distal to the 5’-end (Fig. 4d). Collectively, these data demonstrate that mismatch position critically influences Cas13d activity, with middle and 5′ end mismatches being more disruptive than those at the 3′ end.

### RfxCas13d and HfCas13d induces collateral activity in chicken cells

**Fig. 5:**
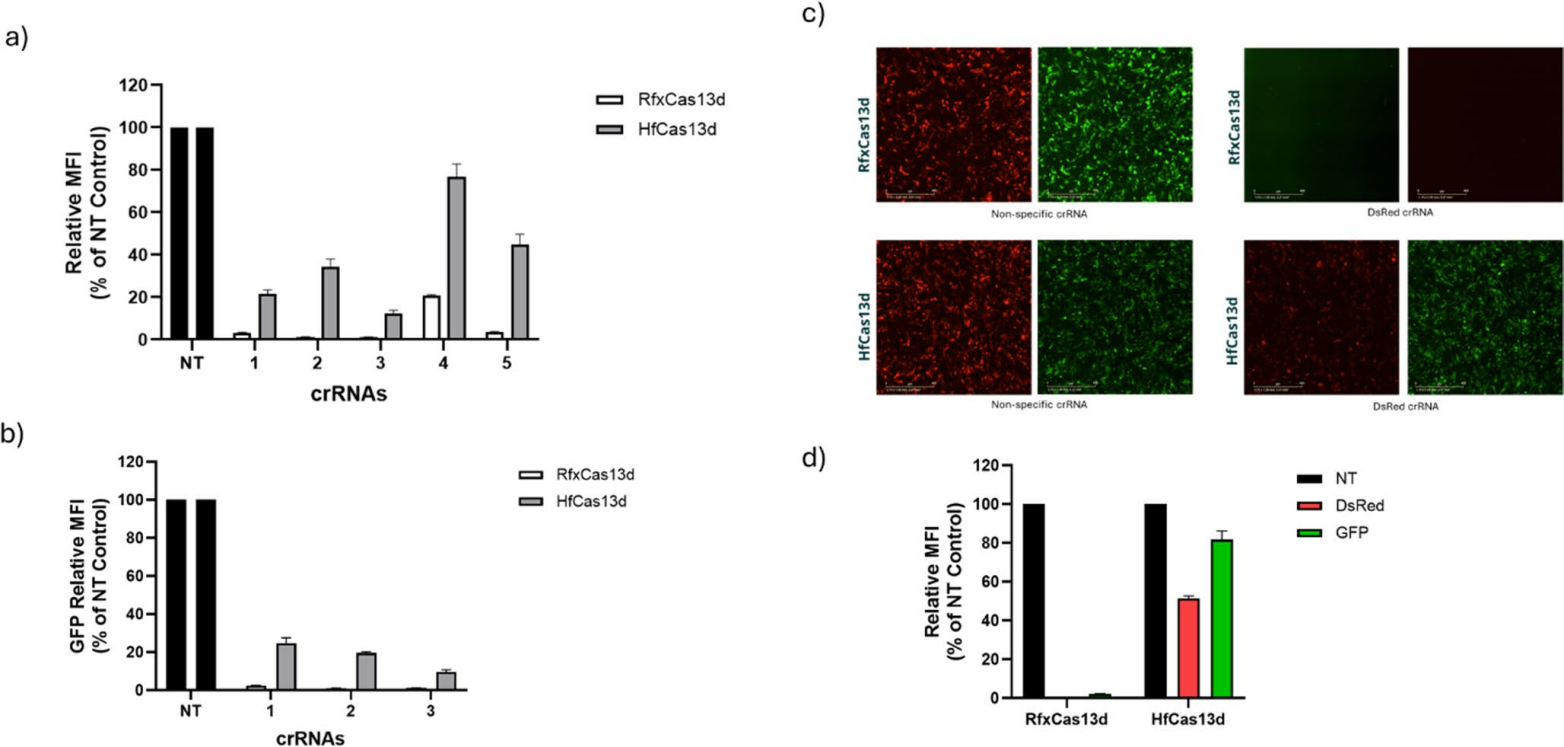
Comparison of on-target and collateral effects of RfxCas13d and HfCas13d in chicken cells. **a** DF1-RfxCas13d and DF1-HfCas13d were each co-transfected with vectors encoding DsRed and five different crRNA (targeting DsRed mRNA) or NT crRNA (negative control). DsRed fluorescence knockdown by different crRNAs was determined by flow cytometry. **b** DF1-RfxCas13d and DF1-HfCas13d were each co-transfected with vectors encoding GFP and individual crRNA (targeting GFP mRNA) or NT crRNAs (negative). The percentage GFP fluorescence knockdown by different crRNAs was determined by flow cytometry. **c** Assessment of collateral activity of RfxCas13d and HfCas13d in chicken cells. The corresponding cell lines were transfected with crRNA targeting DsRed, along with expression vectors for DsRed (on-target) and GFP (serves as collateral reporter). Representative microscopy images of both DsRed and GFP fluorescence degradation in DF1-RfxCas13d cells (top panel) compared with DF1-HfCas13d cells (bottom panel). **d** The percentage DsRed (on-target activity) and GFP (collateral activity) fluorescence knockdown measured by flow cytometry. Values shown as the mean ± Sd. Data points in the graph are averages of the normalised mean fluorescence intensity from technical triplicates

Although RfxCas13d has showed robust mRNA knockdown in our study and in several published reports (Konermann et al. 2018; Kushawah et al. 2020; Zhang et al. 2021), there is substantial evidence in the literature on the collateral activity of Cas13d proteins (Li et al. 2023; Shi et al. 2023). To mitigate this undesired property of RfxCas13d, Tong et al. (2023) developed HfCas13d that reportedly has a reduced collateral activity whilst possessing high on-target cleavage activity. To directly compare the on-target and collateral effects of these two effectors, we established DF1 cell lines stably expressing either RfxCas13d or HfCas13d using Tol2 transposon system (Fig. S1). To assess knockdown activity, RfxCas13d and HfCas13d cells were co-transfected individually with five validated 28-nt DsRed-targeting crRNAs and a DsRed expression vector. Flow cytometry analysis revealed that RfxCas13d consistently showed high levels of DsRed knockdown, in which DsRed crRNAs #1, #2, #3, and #5 exhibiting up to ~ 99% fluorescence knockdown. In contrast, the same crRNAs induced a modest knockdown (~ 50–60%) when targeting DsRed in HfCas13d-expressing cells. Interestingly, crRNA#4, which resulted in ~ 80% DsRed knockdown using RfxCas13d, further diminished its activity in HfCas13d cells, resulting in only ~ 20% knockdown efficiency (Fig. 5a). Given the transgenic DF1 cell lines expressing RfxCas13d and HfCas13d may produce variable expression levels of respective transgenes, we conducted an additional experiment to alleviate any potential variability during characterisation. For this experiment, all three components (Cas13d, corresponding crRNA-DsRed and pCMV-DsRed) were transfected at equal DNA concentration into WT chicken DF1 cells. Fluorescence microscopy analysis of these transfection experiments also revealed that the RfxCas13d cells consistently outperformed knockdown activity across all five DsRed crRNAs (Fig. S6), suggesting that the HfCas13d exhibits reduced targeting efficacy even in cells transfected with equivalent DNA concentration of both variants. The results obtained with five DsRed targeting crRNA transient transfection experiments thus agree with those data obtained in our stable Cas13d-expressing cell lines.

To further verify whether RfCas13d and HfCas13d exhibit variable RNA-targeting efficiency, we selected GFP as an additional target and designed three new 28-nt crRNAs targeting GFP transcripts. Consistent with our observations for DsRed, RfxCas13d consistently outperformed HfCas13d, achieving GFP knockdown levels up to ~ 98–99% across all three crRNAs tested. In contrast, the HfCas13d variant yielded more variable knockdown efficiencies, ranging from 75% to 90% (Fig. 5b & Fig. S7). These data revealed that although HfCas13d mediates RNA targeting, its knockdown efficiency was reduced relative to that of RfxCas13d.

To directly compare the collateral activity of Cas13d effectors, we established a dual-fluorescence reporter assay in which DsRed served as the on-target and GFP as the collateral activity reporter. In this system, successful cleavage (cis) of DsRed mRNA is expected to induce indiscriminate degradation of non-target RNAs (trans), including GFP mRNA. To test this, stable DF1 cells expressing RfxCas13d and HfCas13d were co-transfected with vectors encoding crDsRed#2, or crNT, along with pCMV-DsRed and pCAG-GFP. Fluorescence microscopy analysis revealed that both Cas13d effectors induce on-target and collateral activity, albeit at varied levels (Fig. 5c). In RfxCas13d-expressing cells, sequence-specific targeting of DsRed mRNA led to a substantial reduction (up to ~ 99%) in both DsRed and GFP fluorescence, indicating robust on-target cleavage accompanied by collateral degradation of GFP transcripts. In contrast, HfCas13d-expressing cells displayed only 50% of DsRed fluorescence compared to that was observed in RfxCas13d cells (~ 99%). Furthermore, GFP fluorescence knockdown resulting from collateral activity was largely unaffected with only 10% reduction, suggesting that the collateral activity in HfCas13d cells was significantly attenuated (Fig. 5d, S8). These findings suggest that HfCas13d induces minimal collateral activity, albeit with the trade-off of reduced on-target cleavage activity compared to RfxCas13d.

**Fig. 6:**
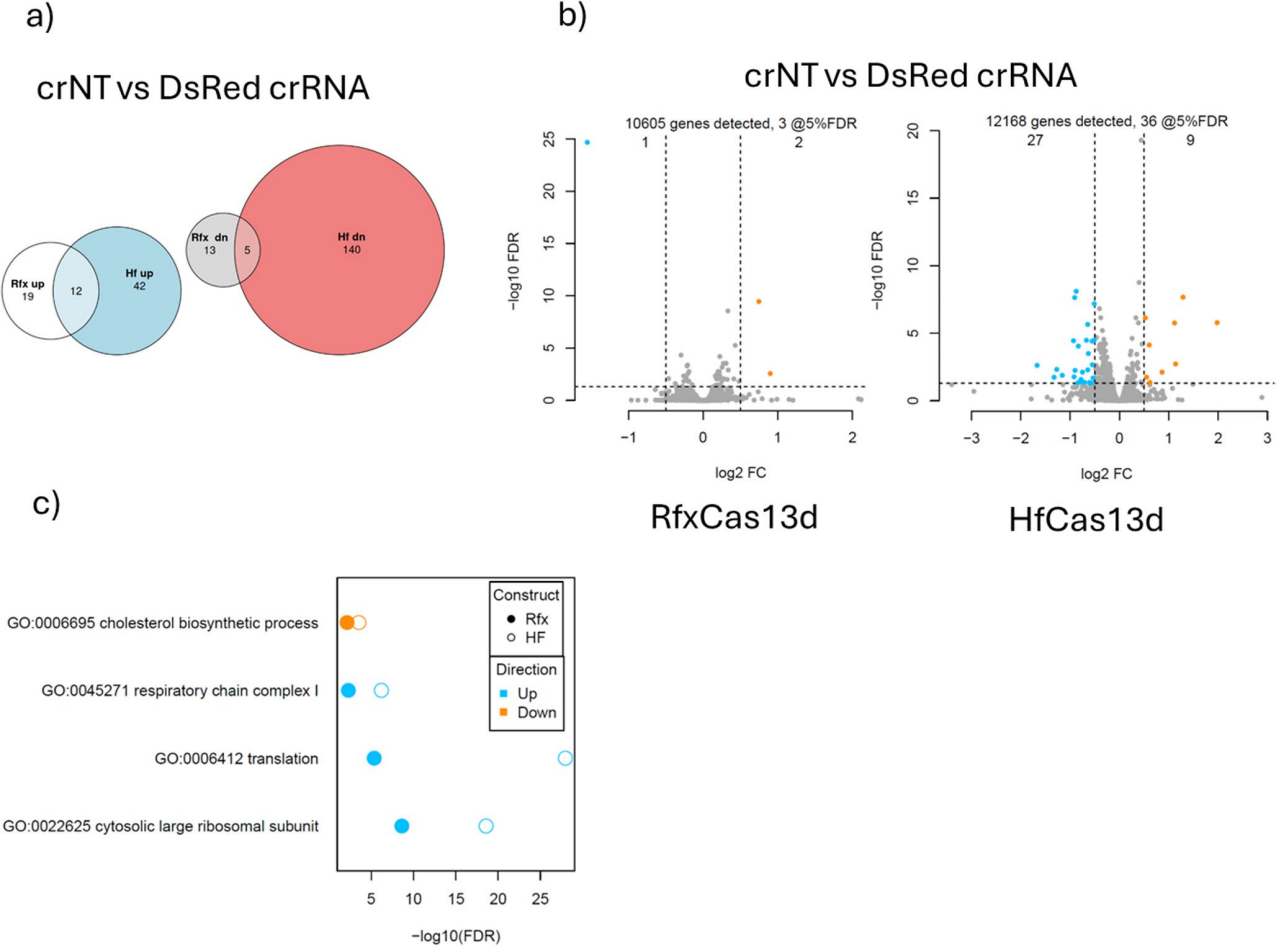
Transcriptome-wide collateral activity of RfxCas13d and HfCas13d in chicken cell lines. **a** Venn diagram of overlapping differentially expressed genes (DEGs) upregulated or downregulated. **b** Volcano plot of the RNA-seq analysis comparing RfxCas13d (left) or HFCas13d (right) DF1 transgenic cells transfected with DsRed and GFP, and either DsRed-crRNA or NT-crRNA. Lines denote a > 1.25-fold change (FC). **c** Gene ontology (GO) and biological process (BP) term analysis for the DEGs in (b)

To examine the downstream effects of Cas13d’s collateral cleavage on endogenous genes, we performed RNA-seq analysis on cells transfected cells with either crDsRed#2, or crNT and both pCMV-DsRed and pCAG-GFP. Compared to the crNT control, catalytically active RfxCas13d perturbed the expression of 49 endogenous genes significantly (FDR < 0.05), whilst HfCas13d disrupted the regulation of 199 genes (Fig. 6a). Despite the presence of these differentially expressed genes (DEGs), only 3 DEGs in RfxCas13d DF1 cells were considered to have had a substantial change in expression (> 1.25 fold-change), compared to the 36 observed in HfCas13d (Fig. 6b). Gene ontology and biological process analysis found that the DEGs belonged in four distinct areas: cholesterol biosynthetic process, respiratory chain complex I, translation and the cytosolic large ribosomal subunit (Fig. 6c). Despite the presence of these DEGs, and their connection to key biological processes, the expression changes of individual genes in these pathways were determined to be quite minor and have limited effect on cell viability.

### RfxCas13d mediated knockdown of NP gene of H5N1 2.3.4.4b

**Fig. 7:**
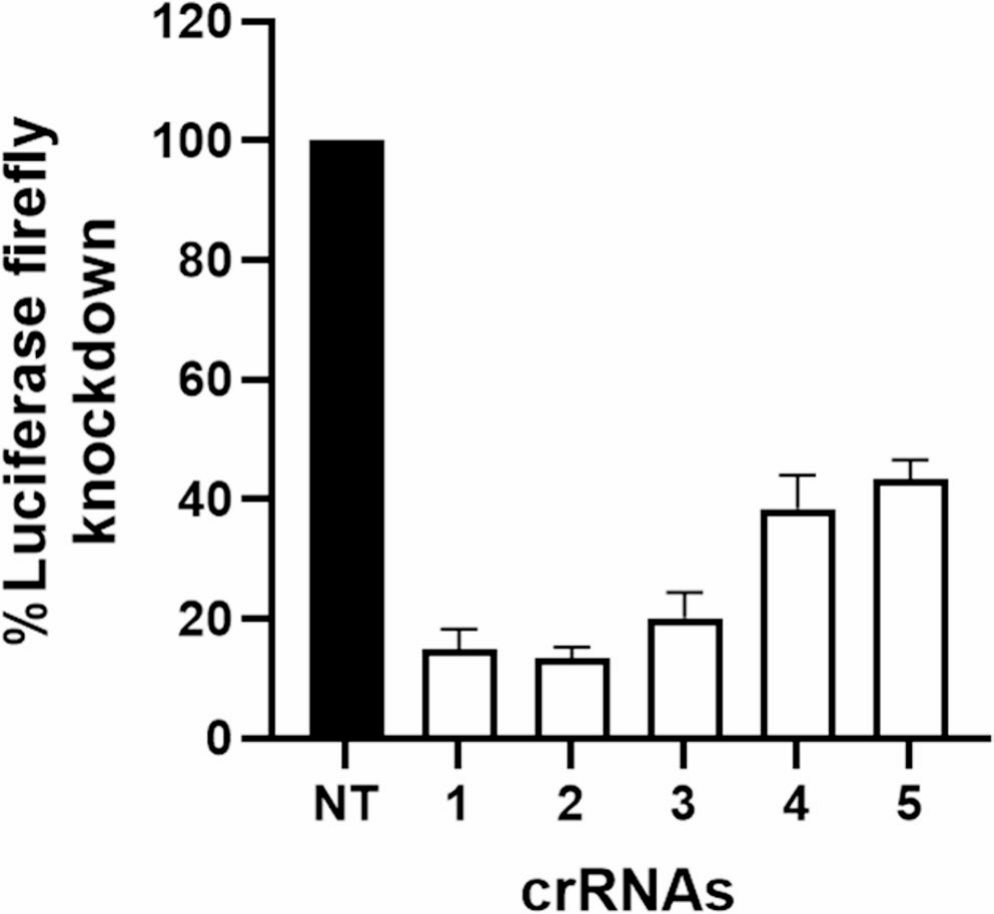
RfxCas13d mediated knockdown of nucleoprotein mRNA of avian influenza virus. Five spacers targeting distinct regions within the NP gene segment of A/Turkey/Indiana/22–003707-003/2022/H5N1 (Clade: 2.3.4.4b). Knockdown of H5N1 NP gene by transfection of individual crRNAs and siCHECK-2 + NP into RfxCas13d cells. Firefly luciferase activity measured and represented as percentage of inhibition relative to NT crRNA

To investigate whether RfxCas13d can be repurposed to target synthetic sequences of viral RNA, we selected the nucleoprotein (NP) gene of H5N1 clade 2.3.4.4b influenza A strain, A/turkey/Indiana/3703-003/2022. To quantitatively measure the viral RNA knockdown, a luciferase reporter construct was generated by fusing firefly luciferase in-frame with the 200-bp NP target region. Five crRNAs (crNP#1–crNP#5; Supplementary Table 1) were designed across the NP sequence. The luc-NP and crRNA encoding plasmids were then co-transfected into RfxCas13d expressing cell lines. Luciferase assay revealed that crNP#1 and crNP#2 achieved knockdown efficiencies of approximately 86% and 87%, respectively, whereas crNP#3 mediated a ~ 79% knockdown. In contrast, crNP#4 and crNP#5 showed reduced knockdown efficiencies of ~ 61% and ~ 56%, respectively (Fig. 7).

## Discussion

Efficient target basepairing and nuclease activation of the Cas13-crRNA complex require a stable crRNA-target interaction. In theory, any crRNA that maintains complete complementarity of 28-nt to the target RNA is expected to mediate functional knockdown. Using 13 distinct crRNAs tested against three targets (DsRed, GFP and NP genes), we found that not all crRNAs were capable of mediating mRNA knockdown. Indeed, previous studies have revealed that a single nucleotide shift in the crRNA position profoundly impacts Cas13 functional activity (Hu et al. 2024). This variability in knockdown activity is partly attributed to factors such as RNA structure, spacer accessibility, thermodynamics at spacer–target interface, spacer sequence, and other, unknown features. Our DsRed knockdown assays using truncated crRNAs revealed that a 24-nt crRNA was as efficient as a 28-nt crRNA, albeit truncations beyond 24-nt led to a stepwise decrease in knockdown efficiency and a near-complete loss of activity with a 16-nt spacer (Fig. 4c and d). This minimal spacer length threshold likely reflects the structural requirements for an efficient spacer–target duplex and the necessary conformational changes to activate HEPN domains within the Cas13d + crRNA + target complex (Zhang et al. 2018, 2019a, b; Konermann et al. 2018). Similar truncation studies on oncogenic mutant targets and RfxCas13d in human cells revealed a crRNA length dependency, in which 24-nt crRNA induced effective knockdown (Wessels et al. 2024). Furthermore, our results are in agreement with those of two other studies by Zhang et al. (2018) and Bandaru et al. (2020), the latter of which identified the region between nucleotides 11 and 18 within the crRNA as critical for target recognition. Based on the cumulative knockdown efficiency using 21-nt crRNA, in which the DsRed fluorescence levels (i.e., MFI) were similar to those achieved using 28-nt crRNAs (Fig. 3d), we believe that the use of shorter spacer sequences is possible in specific applications. For example, in applications involving the transgenic expression of Cas13d and crRNAs, it is expected that the trade-off on knockdown activity using truncated crRNAs is minimal, owing to the abundant Cas13 + crRNA-target stoichiometry intracellularly resulting from transgenic expression. This is of particular interest for antiviral interventions against rapidly mutating RNA viruses, in which shorter spacers provide broader coverage of viral subtypes. However, one major limitation of using shorter spacers is their reduced specificity compared to that of longer crRNAs, as the likelihood of targeting partially matched homologous sequences could be exacerbated.

Owing to their evolutionary origin as an adaptive immune system, CRISPR-Cas systems are known to exhibit a degree of mismatch tolerance at the crRNA-target interface. This flexibility has been observed in both DNA-targeting and RNA-targeting Cas effectors, and may be advantageous for antiviral interventions. However, where the emergence of mutants is a common phenomenon, it may compromise the efficacy or safety of the system in applications that require targeting at single-nt precision. Using our best performing 24-nt truncated crRNA, we revealed that the Cas13d mismatch tolerance was primarily influenced by two aspects: (i) the number of mismatches within the crRNA-target and (ii) the position at which these mismatches are introduced into crRNA. Regardless of the position at which single-nucleotide mismatch was introduced within the crRNA, knockdown activity was largely not affected (Fig. 4b). It is also interesting to note that impact on knockdown activity was unaffected even with a 4-nt mismatch introduced at the 3’ end of the crRNA. On the other hand, even a 2-nt or 3-nt mismatch at the 5’ or middle regions have substantial impact, and a 4-nt mismatch completely abolished cleavage activity (Fig. 4b). These findings are in agreement with previous studies on RfxCas13d (Chen et al. 2025) and other Cas13 orthologs Cas13a (Tambe et al. 2018; Molina Vargas et al. 2024) Cas13b (Fareh et al. 2021; Hu et al. 2024). This differential response of Cas13d activity for the introduced mismatches within the middle and 5’-end of the crRNA can be attributed to impact on thermodynamic stability of the crRNA-RNA duplex. Such perturbations often impair crRNA-target interaction and attenuates the Cas13d cleavage activity (Wessels et al. 2024). Additionally, given the critical role of the 11 nt to 18 nt (mid) position within crRNA for proper binding (Tambe et al. 2018), and the fact that the 5’ end is close to PFS base, mismatches within this position affect the Cas13d structural conformation for cleavage. Indeed, single-nt mismatches introduced at the 1 st, 2nd, 3rd and 4th base indicate that mismatches distal to the PFS exert minimal functional disruption (Fig. 4c). The findings from this study provide new insights into the functional tolerance of RfxCas13d at the crRNA-target interface and suggesting the possibility for rational design of crRNAs that remain effective across diverse and evolving pathogenic mutations.

It is widely acknowledged that most CRISPR-Cas13d systems induce collateral cleavage following target recognition and cleavage. Therefore, understanding the extent of on-target and collateral activity of Cas13 variants in species-specific cell lines is of paramount importance for their translational utility. In our study, direct comparison of knockdown efficiencies induced by RfxCas13d and HfCas13d using eight distinct crRNAs (5 against DsRed and 3 against GFP) confirmed that RfxCas13d exhibits high targeting efficiency (Fig. 5a.b). Furthermore, our dual fluorescence reporter assay has shown reduced collateral activity in HfCas13d transfected cells, albeit at the compromise of the on-target activity (Fig. 5c). The reduced on-target activity observed with HfCas13d in chicken cells is consistent with recent reports in other cell types (Chen et al. 2025; Hart et al. 2025). A possible explanation why RfxCas13d exhibits stronger on-target (i.e., DsRed knockdown) activity than HfCas13d may be in part a result of its high collateral activity. To this end, the observed suppression of DsRed might not reflect just precise and sequence-specific targeting alone, but rather a combination of both on-target and collateral cleavage. Disentangling these questions can be challenging in cell culture systems, and therefore thorough biochemical analyses are needed to distinguish true on-target effects from collateral activity in both RfxCas13d and HfCas13d systems.

RNAseq analysis on collateral activity revealed that only three endogenous genes were significantly dysregulated by RfxCas13d. This limited collateral cleavage of endogenous genes is corroborated by previous reports with other Cas13 orthologs, in which limited to no collateral targeting is observed (Abudayyeh et al. 2017; Tieu et al. 2024). However, further research may be required to investigate if targeting viral genes during infection will produce the same result. It is also unknown why HfCas13d had increased collateral activity in endogenous genes compared to RfxCas13d. Further exploration on whether this effect is consistent across multiple targeting systems could prove valuable. The induction of collateral activity in this study was mediated by targeting an exogenous transcript (i.e., DsRed mRNA), which may inherently limit the extent of transcriptome-wide perturbation. This is likely because exogenous targets differ from endogenous transcripts in their abundance, localisation, and association with native RNA-binding proteins. We acknowledge that this approach may not fully capture the cumulative impact of collateral activity. Therefore, additional studies using cell lines that stably express respective Cas13d variants and crRNAs targeting endogenous transcript will be critical. Such studies may provide a basis for our understanding on the degree of collateral degradation and its consequences. These studies will be critical to elucidate Cas13d-induced transcriptomic perturbations under stable expression and inform the safety profile for therapeutic and biotechnological applications.

## Supplementary Information

Below is the link to the electronic supplementary material.

Supplementary Material 1

Supplementary Material 2

## Data Availability

RNA-seq data has been deposited to NCBI GEO and is available under accession number GSE305666 (https://www.ncbi.nlm.nih.gov/geo/query/acc.cgi? acc=GSE305666).
