# Supplementary material for: Systematic evaluation of CrRNA design parameters for optimized Cas13d-mediated RNA targeting in chicken cells
Source: Funct Integr Genomics. 2025 Nov 26;25(1):256. doi: 10.1007/s10142-025-01776-x (PMC12647337; doi:10.1007/s10142-025-01776-x)
Supplement: Supplementary file 2 — Supplementary Material 2 [file 10142_2025_1776_MOESM2_ESM.pdf]

OFFICIAL

Systematic Evaluation of crRNA Design Parameters for Optimized Cas13d-Mediated RNA Targeting in Chicken Cells

Functional & Integrative Genomics

Emily Hann[1,2], Debolina Majumdar[1], Daniel Layton[1], Mohamed Fareh[4], David M. Cahill[2], Mark Ziemann[3], Beata Ujvari[2], Karel A Schat[5], Arjun Challagulla*[1]

[1]Australian Centre for Disease Preparedness, CSIRO Health and Biosecurity, Geelong, VIC, Australia,

[2]School of Life and Environmental Sciences, Deakin University, Geelong, VIC, Australia

[3]Burnet Institute, Melbourne, VIC, Australia

[4]Peter MacCallum Cancer Centre, Melbourne, VIC, Australia

[5]Department of Microbiology and Immunology, College of Veterinary Medicine, Cornell University, Ithaca, NY, USA.

*Corresponding author: Arjun Challagulla (Arjun.Challagulla@csiro.au)

OFFICIAL

Supplementary Table 1. List of crRNAs used in the study

**Target**	**crRNA name**	**Sequence (5’ – 3’)**
Non-specific	crNT	ATGCATGCATGCATGCATGCATGCATGC
DsRed	crDsRed#1	CCGTTCACGGAGCCCTCCATGCGCACCT
DsRed	crDsRed#2	GGACAGCTTCTTGTAGTCGGGGATGTCG
DsRed	crDsRed#3	CAGGCGCTCGGTGGAGGCCTCCCAGCCC
DsRed	crDsRed#4	GTCCAGCTTGGAGTCCACGTAGTAGTAG
DsRed	crDsRed#5	TCGAGATCTCTGGAACAGGTGGTGGCGG
DsRed	crDsRed28-A-PFS#1	TCTTGTAGTCGGGGATGTCGGCGGGGTG
DsRed	crDsRed28-G-PFS	AGCTTCTTGTAGTCGGGGATGTCGGCGG
DsRed	crDsRed28-C-PFS	GACAGCTTCTTGTAGTCGGGGATGTCGG
DsRed	crDsRed28-A-PFS#2	AGTCGGGGATGTCGGCGGGGTGCTTCAC
DsRed	crDsRed28-A-PFS#3	GTAGTCGGGGATGTCGGCGGGGTGCTTC
DsRed	crDsRed28-A-PFS#4	CTTGTAGTCGGGGATGTCGGCGGGGTGC
DsRed	crDsRed24	GGACAGCTTCTTGTAGTCGGGGAT
DsRed	crDsRed23	GGACAGCTTCTTGTAGTCGGGGA
DsRed	crDsRed22	GGACAGCTTCTTGTAGTCGGGG
DsRed	crDsRed21	GGACAGCTTCTTGTAGTCGGG
DsRed	crDsRed20	GGACAGCTTCTTGTAGTCGG
DsRed	crDsRed16	GGACAGCTTCTTGTAG
DsRed	crDsRed-5’-1nt	CGACAGCTTCTTGTAGTCGGGGAT
DsRed	crDsRed-5’-2nt	CCACAGCTTCTTGTAGTCGGGGAT
DsRed	crDsRed-5’-3nt	CCTCAGCTTCTTGTAGTCGGGGAT
DsRed	crDsRed-5’-4nt	CCTGAGCTTCTTGTAGTCGGGGAT
DsRed	crDsRed-5’-8nt	CCTGTCGATCTTGTAGTCGGGGAT
DsRed	crDsRed-5’-12nt	CCTGTCGAAGAAGTAGTCGGGGAT
DsRed	crDsRed-Mid-1nt	GGACAGCTTCTTCTAGTCGGGGAT
DsRed	crDsRed- Mid -2nt	GGACAGCTTCTACTAGTCGGGGAT
DsRed	crDsRed- Mid -3nt	GGACAGCTTCAACTAGTCGGGGAT
DsRed	crDsRed- Mid -4nt	GGACAGCTTCAACAAGTCGGGGAT
DsRed	crDsRed- Mid -8nt	GGACAGCTAGAACATCTCGGGGAT
DsRed	crDsRed- Mid -12nt	GGACAGGAAGAACATCAGGGGGAT
DsRed	crDsRed-3’-1nt	GGACAGCTTCTTGTAGTCGGGGAA
DsRed	crDsRed-3’-2nt	GGACAGCTTCTTGTAGTCGGGGTA
DsRed	crDsRed-3’-3nt	GGACAGCTTCTTGTAGTCGGGCTA
DsRed	crDsRed-3’-4nt	GGACAGCTTCTTGTAGTCGGCCTA
DsRed	crDsRed-3’-8nt	GGACAGCTTCTTGTAGAGCCCCTA
DsRed	crDsRed-3’-12nt	GGACAGCTTCTTCATCAGCCCCTA
DsRed	crDsRed-5’-1nt-2ndbase	GCACAGCTTCTTGTAGTCGGGGAT
DsRed	crDsRed-5’-1nt-3rdbase	GGTCAGCTTCTTGTAGTCGGGGAT
DsRed	crDsRed-5’-1nt-4thbase	GGAGAGCTTCTTGTAGTCGGGGAT
GFP	crGFP#1	TTACGTCGCCGTCCAGCTCGACCAGGAT
GFP	crGFP#2	GTCCTCCTTGAAGTCGATGCCCTTCAGC
GFP	crGFP#3	GGCGGACTGGGTGCTCAGGTAGTGGTTG
NP IAV gene	crNP#1	GGATCGTTTGGTGCCTTGAGACGC
NP IAV gene	crNP#2	GTCTCCATCTGTTCATAGGATCGT
NP IAV gene	crNP#3	CAACCATTCTTCCAACAGATGCTC
NP IAV gene	crNP#4	CACATCTGTATGTAGAACCTCCCA
NP IAV gene	crNP#5	TATTGTTATGCTGTTCTGGATCAG

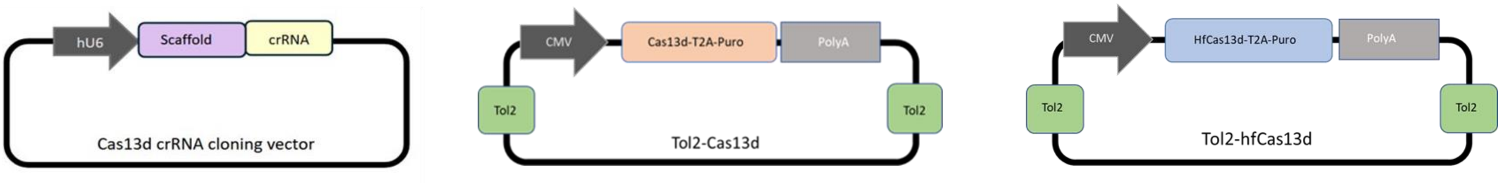

Figure S1. Schematics representation of crRNA expression plasmid, and Tol2 transposon vectors carrying RfxCas13d or HfCas13d.

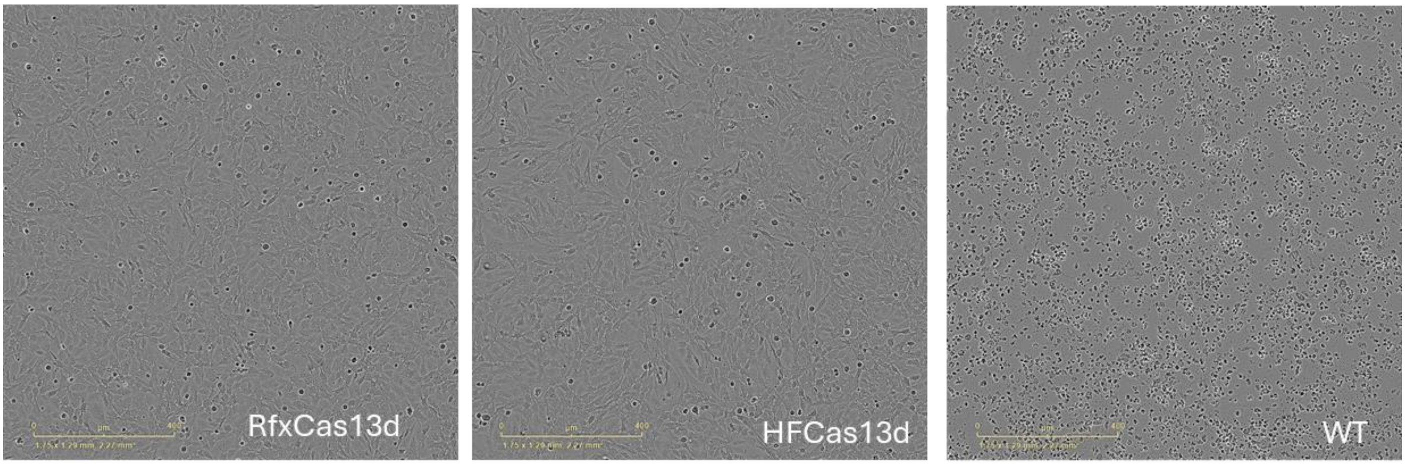

Figure S2. Bright field images of stable DF1-RfxCas13d and DF1-HfCas13d cells generated by puromycin selection. Cells were treated with 1.2 ug/mL puromycin for 14 days to isolate stably integrated cell lines. Wild-type (WT) DF1 cells were used as a negative control for puromycin selection.

OFFICIAL

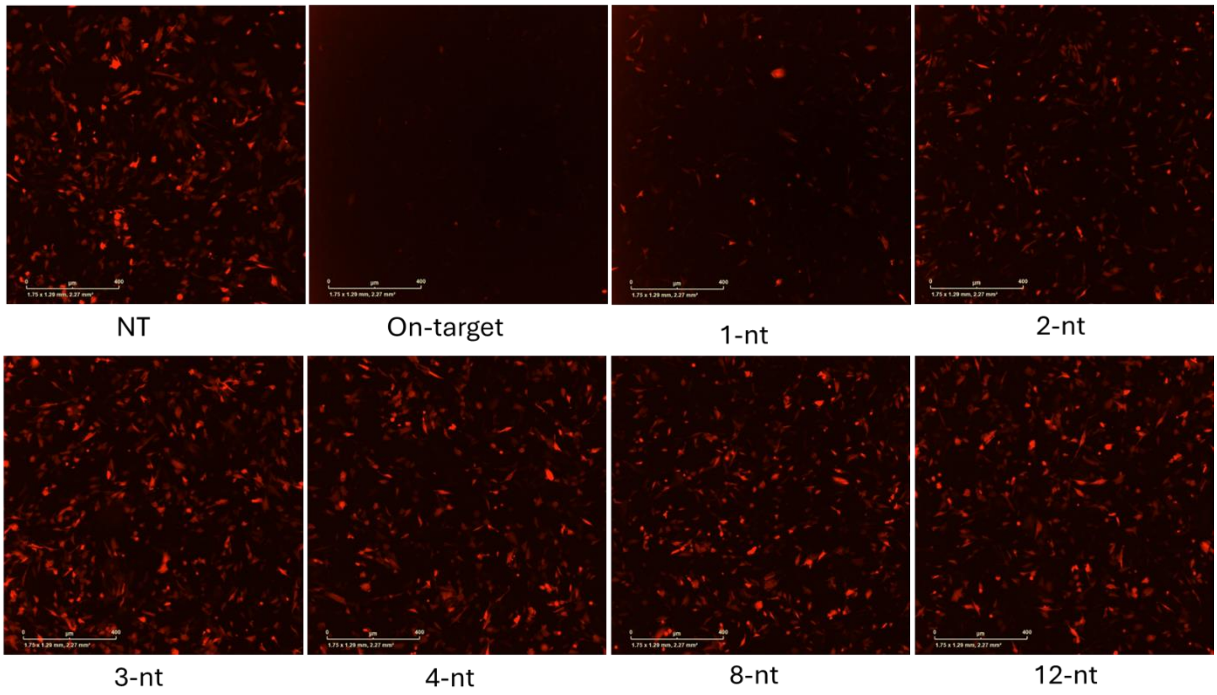

Figure S3. Representative fluorescence microscopy images show the DsRed knockdown of crRNAs with mismatches introduced at the 5' end of the spacer.

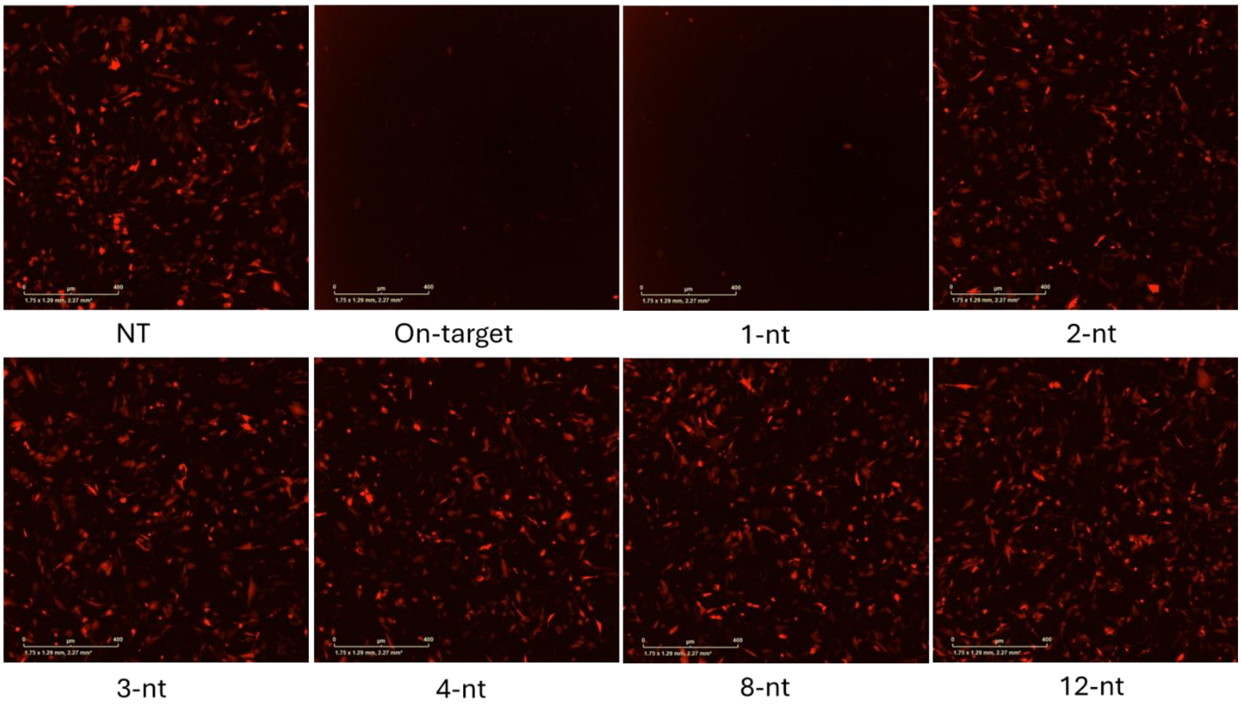

Figure S4. Representative fluorescence microscopy images show the DsRed knockdown of crRNAs with mismatches introduced in the middle region of the spacer.

OFFICIAL

OFFICIAL

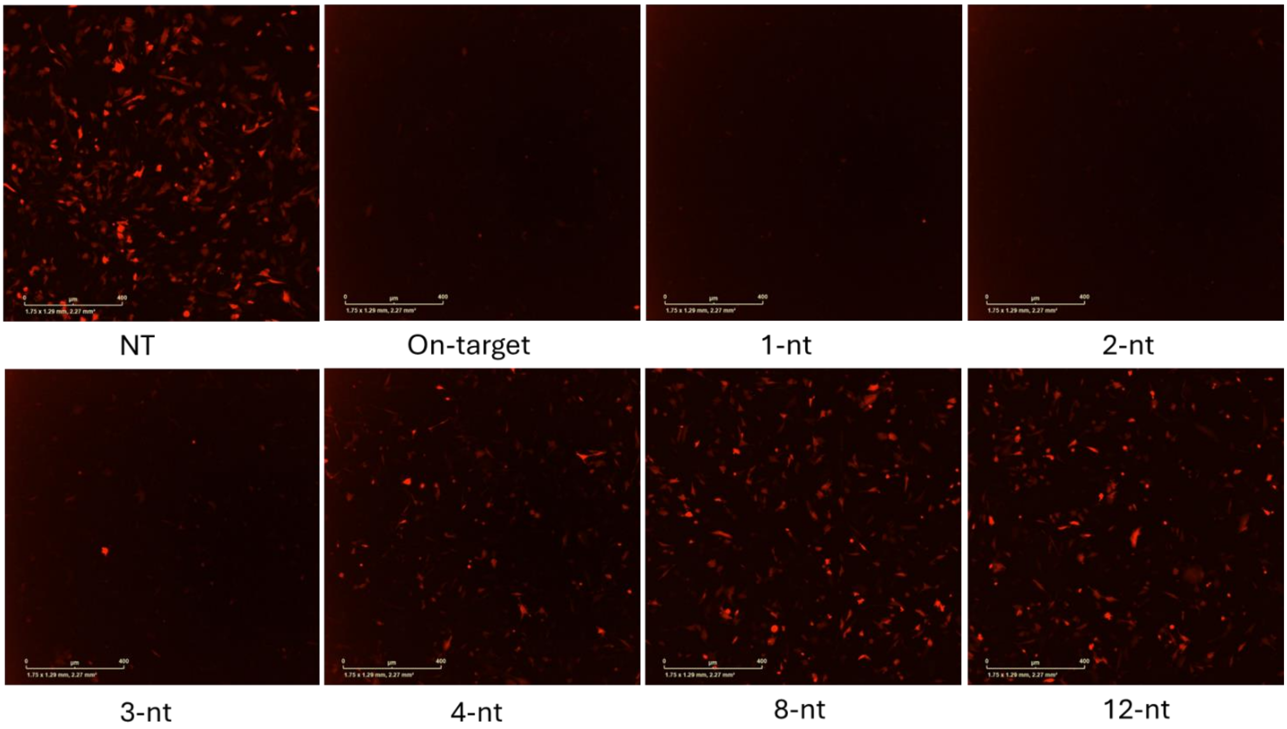

Figure S5. Representative fluorescence microscopy images show the DsRed knockdown of crRNAs with mismatches introduced at the 3’ end of the spacer.

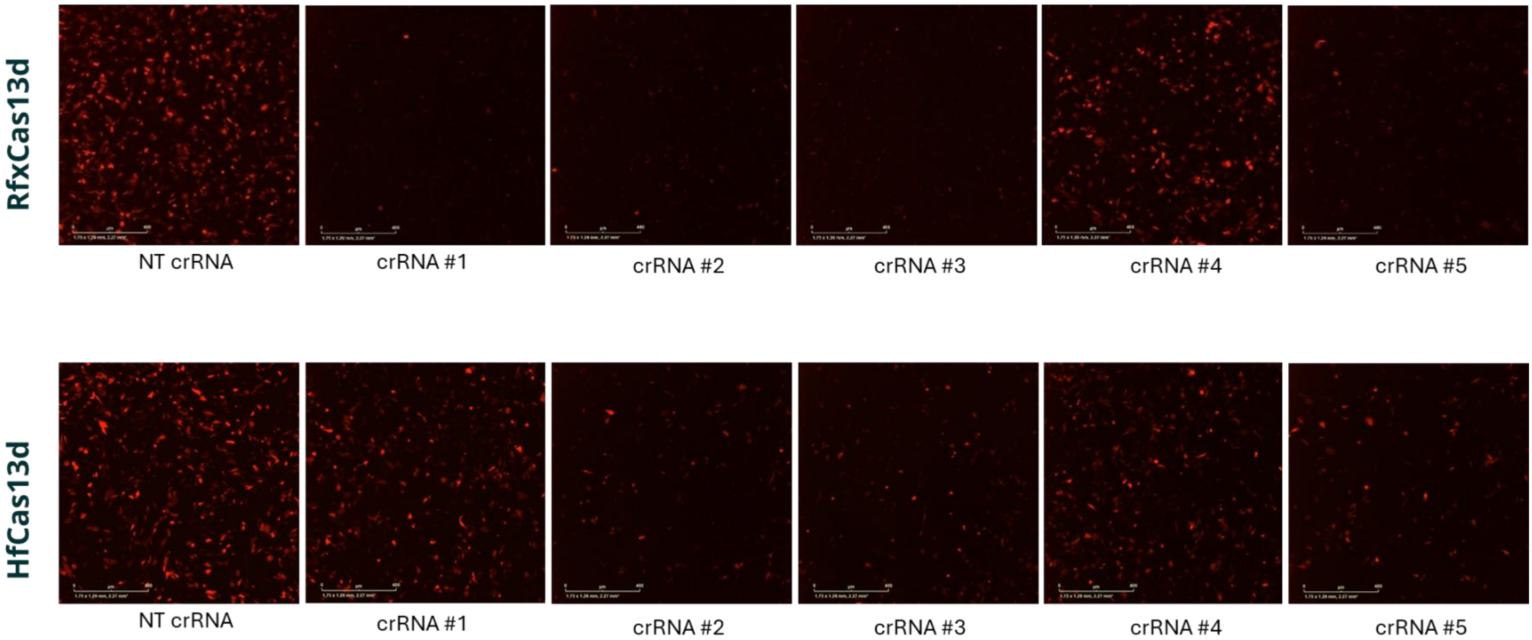

Figure S6. Representative fluorescence microscopy images comparing RfxCas13d and HFCas13d targeting of DsRed mRNA using transient transfection of Cas13d, crRNA and DsRed vectors in WT DF1 cells.

OFFICIAL

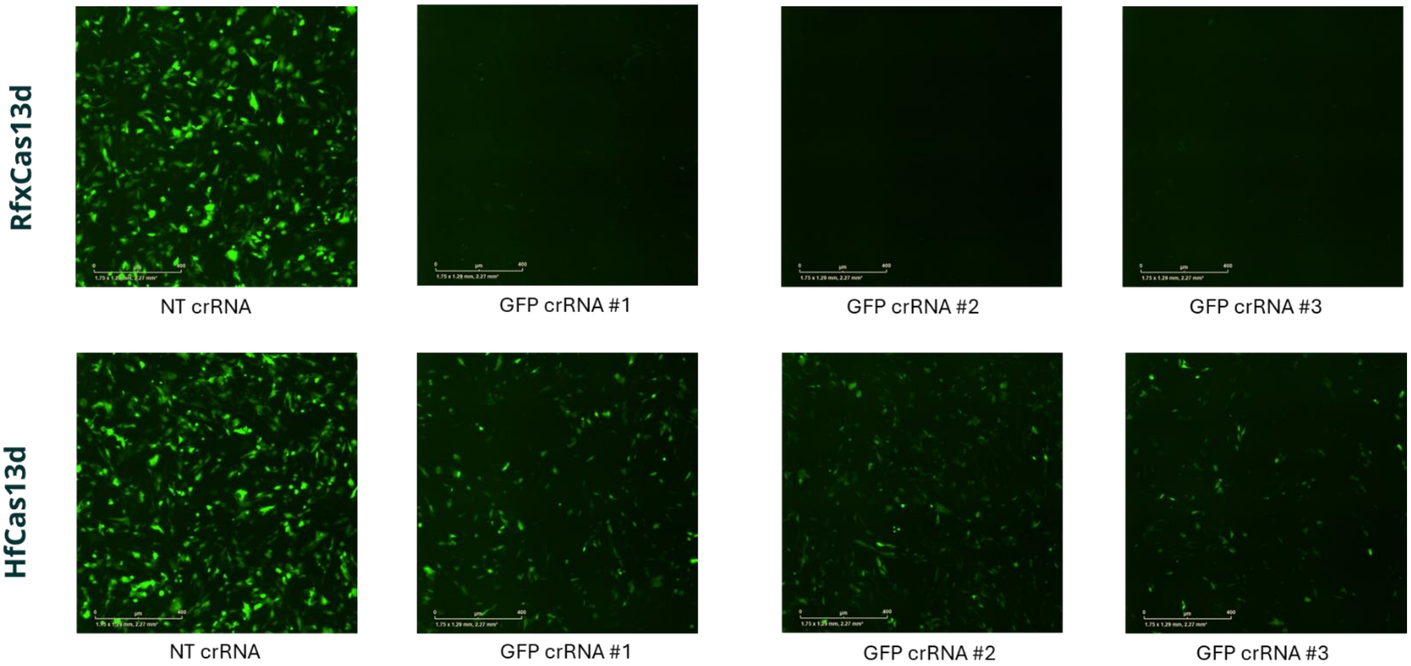

Figure S7. Representative fluorescence microscopy images comparing RfxCas13d and HfCas13d knockdown activity using GFP as the target.
